# Development and validation of a clinical prediction model for postoperative atrial fibrillation after lung cancer surgery: a machine-learning–based study

**DOI:** 10.3389/fsurg.2026.1793385

**Published:** 2026-06-04

**Authors:** Yi Xu, Ting Lu, Ke Xu, Xiaoyan Feng, Rongsheng Xiong

**Affiliations:** 1 Department of Thoracic Surgery, Nanxishan Hospital of Guangxi Zhuang Autonomous Region (The Second People's Hospital of Guangxi Zhuang Autonomous Region), Guilin, China; 2 Department of Community Health, Pusat Kanser Tun Abdullah Ahmad Badawi, Universiti Sains Malaysia (USM), Penang, Malaysia; 3 Day Chemotherapy Center, The First Affiliated Hospital of Guangxi Medical University, Nanning, China

**Keywords:** logistic regression, lung cancer surgery, machine learning, nomogram, postoperative atrial fibrillation, risk prediction

## Abstract

**Background:**

Postoperative atrial fibrillation (POAF) is a common complication after lung cancer surgery, associated with increased morbidity and prolonged hospitalisation. Accurate preoperative or early postoperative risk stratification remains challenging due to the multifactorial nature of POAF. This study aimed to develop and validate machine learning–based prediction models for POAF and to construct a clinically applicable nomogram for individualised risk estimation.

**Methods:**

A total of 540 patients undergoing lung cancer surgery were retrospectively included, among whom 107 (19.8%) developed POAF. Patients were randomly divided into a training cohort (*n* = 379) and an independent test cohort (*n* = 161). Least absolute shrinkage and selection operator (LASSO) regression with 10-fold cross-validation was applied in the training cohort to select the most informative predictors. Seven machine-learning models—logistic regression (LR), k-nearest neighbours (KNN), decision tree (DT), random forest (RF), extreme gradient boosting (XGBoost), support vector machine (SVM), and neural network (NN)—were developed using the selected features. Model performance was evaluated in both cohorts in terms of discrimination, calibration, and decision curve analysis. A nomogram was constructed based on the optimal model.

**Results:**

LASSO regression identified six predictors of POAF: age, education level, hypertension, marital status, postoperative pain score, and surgical approach. In the training cohort, all models demonstrated good discrimination with area under the receiver operating characteristic curve (AUC) values ranging from 0.827 to 0.995. However, performance declined to varying degrees in the test cohort. LR exhibited the most stable performance, achieving the highest AUC (0.855) and accuracy (0.857), with acceptable precision (0.667), recall (0.563), and F1 score (0.610). Calibration curves indicated good agreement between predicted and observed POAF risks for the LR model, while decision curve analysis demonstrated a consistently favourable net benefit across clinically relevant threshold probabilities. Based on these findings, an LR-based nomogram incorporating the six selected predictors was developed to facilitate individualised POAF risk prediction.

**Conclusions:**

We developed and internally validated a machine learning–assisted risk prediction framework for POAF after lung cancer surgery. Compared with more complex models, LR demonstrated superior stability, calibration, and clinical utility. The resulting nomogram provides a practical and interpretable tool for early postoperative POAF risk assessment and may support perioperative monitoring and personalised management of patients undergoing lung cancer surgery.

## 1 Introduction

Postoperative atrial fibrillation (POAF) is a common cardiac complication after thoracic surgery, particularly following lung cancer resection, with reported incidence rates ranging from 2%–20% (1). Although POAF is often transient, it is associated with adverse clinical outcomes including haemodynamic instability, thromboembolic events, stroke, heart failure, prolonged hospitalisation, higher healthcare costs, and increased postoperative mortality (2, 3). Early identification of patients at high risk of POAF is therefore essential for optimising perioperative monitoring and preventive strategies (4).

Current approaches to POAF risk assessment rely mainly on individual clinical factors such as advanced age, cardiovascular comorbidities, and the invasiveness of surgery (5, 6). Several risk models have been developed, primarily in cardiac surgery populations (7–9); however, their applicability to lung cancer surgery is limited due to differences in patient characteristics, surgical stress, and perioperative management. Moreover, many existing models include a restricted number of predictors and fail to account for complex interactions among demographic factors, perioperative variables, inflammatory responses, and cardiac structural or functional parameters (10). Consequently, reliable surgery-specific prediction tools for POAF after lung cancer resection remain lacking.

Machine-learning (ML) techniques have recently attracted increasing attention in perioperative risk prediction because of their ability to integrate multidimensional clinical data and model non-linear relationships among predictors (11). Prior studies have shown that ML-based models can outperform traditional statistical methods in predicting postoperative complications (12, 13). Nevertheless, highly complex models are prone to overfitting and often lack interpretability, which may limit their clinical applicability and acceptance by clinicians. In the perioperative setting, an ideal prediction model should achieve a balance between discrimination, calibration, robustness, and interpretability to support individualised clinical decision-making.

Therefore, the aim of this study was to develop and validate a prediction model for POAF in patients undergoing lung cancer surgery using routinely available perioperative data. Multiple ML algorithms were systematically compared, and the most clinically robust model was selected based on discrimination, calibration, and clinical utility. Using predictors identified by least absolute shrinkage and selection operator (LASSO) feature selection, an interpretable nomogram was constructed to facilitate individualised POAF risk estimation and to support perioperative risk stratification and personalised management. This approach addresses the current lack of dedicated POAF prediction tools for lung surgery patients and leverages machine-learning techniques to potentially improve risk stratification in this population.

## 2 Materials and methods

### 2.1 Study design and data source

**Figure 1:**
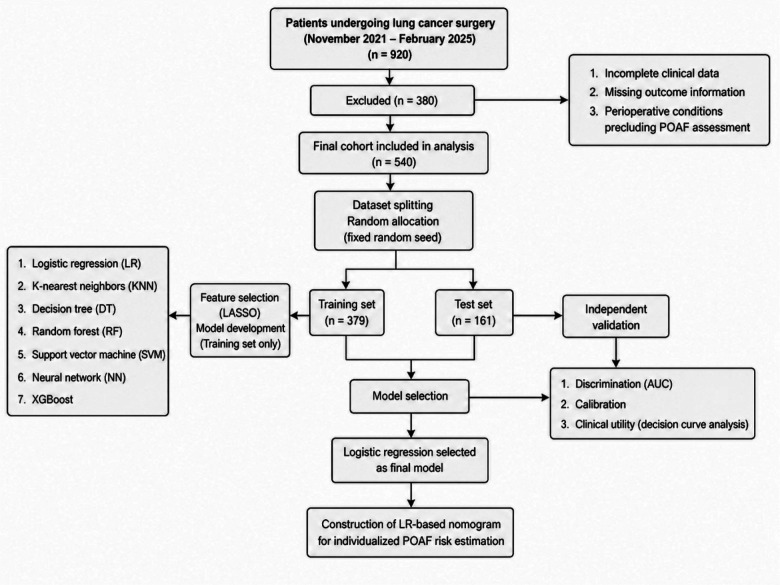
Flowchart of patient selection, dataset splitting, model development, and validation. A total of 920 patients undergoing lung cancer surgery were screened, of whom 540 were included in the final analysis. Patients were randomly divided into a training cohort (*n* = 379) and an independent test cohort (*n* = 161). The training cohort was used for feature selection and model development, and the test cohort was used for model validation and performance evaluation.

This retrospective observational study was conducted at Nanxishan Hospital of Guangxi Zhuang Autonomous Region, a tertiary referral centre in China. Consecutive patients who underwent surgical treatment for lung cancer between November 2021 and February 2025 were screened for eligibility. A total of 920 patients were initially identified. Patients with a documented history of atrial fibrillation or atrial flutter before surgery, evidence of atrial fibrillation or atrial flutter on preoperative electrocardiography, incomplete clinical data, missing outcome information, or perioperative conditions precluding accurate POAF assessment were excluded. Only patients who were in sinus rhythm before surgery were included. Intraoperative cardiac rhythm monitoring records were also reviewed when available to ensure that POAF represented a new-onset postoperative arrhythmic event rather than the continuation or recurrence of a prior atrial arrhythmia. Ultimately, 540 patients were included in the final analysis. POAF was defined as newly diagnosed atrial fibrillation occurring during the postoperative hospital stay, confirmed by standard electrocardiography or continuous cardiac monitoring. The overall study workflow, including patient selection, exclusion criteria, dataset splitting, feature selection, model development, validation, and clinical translation, is summarised in Figure 1.

### 2.2 Ethical approval

This study was approved by the Ethics Committee of Nanxishan Hospital of Guangxi Zhuang Autonomous Region (Approval No. NXSYY-2024-214). Owing to the retrospective nature of the study and the use of anonymised clinical data, the requirement for informed consent was waived by the ethics committee. The study was conducted in accordance with the Declaration of Helsinki and relevant institutional guidelines.

### 2.3 Outcome and candidate predictors

The primary outcome was the occurrence of POAF. POAF was defined as new-onset atrial fibrillation occurring during the postoperative hospital stay, from postoperative day 0 to discharge, lasting at least 30 s and documented by standard 12-lead electrocardiography or continuous electrocardiographic monitoring. Postoperative rhythm monitoring was performed according to institutional routine practice. Patients routinely underwent continuous bedside electrocardiographic monitoring during the early postoperative period, generally for 24–72 h depending on clinical condition, surgical complexity, and perioperative risk. After discontinuation of continuous monitoring, additional 12-lead electrocardiography or rhythm assessment was performed when patients developed symptoms such as palpitations, chest discomfort, dyspnoea, haemodynamic instability, or abnormal pulse/rhythm findings during routine ward observation. All POAF episodes detected during the postoperative hospital stay were recorded.

Candidate predictors were selected *a priori* based on clinical relevance and existing literature, and included demographic characteristics, comorbidities, perioperative variables, laboratory parameters, inflammatory markers, cardiac functional indices, and postoperative pain scores. All candidate predictors were collected preoperatively or during the early postoperative period before the occurrence of POAF. Accordingly, the resulting model was intended for early postoperative risk stratification rather than purely preoperative prediction. Postoperative pain score was assessed using the Numeric Rating Scale after recovery from anaesthesia during the early postoperative period. For patients who developed POAF, the earliest available pain score recorded before the onset or diagnosis of POAF was used. Pain scores recorded after POAF onset were not used as predictors. For patients without POAF, the corresponding early postoperative pain score recorded during routine postoperative assessment was used. In this study, lung cancer surgery referred to therapeutic pulmonary resection performed for patients with lung cancer. According to operative records, surgical approach was classified as video-assisted thoracoscopic surgery (VATS) or open thoracotomy. The extent of resection included wedge resection, segmentectomy, and lobectomy. Surgical approach and extent of resection were extracted from medical records as perioperative candidate variables because they may reflect differences in surgical invasiveness, operative stress, and POAF risk. Continuous variables included age, postoperative pain score, laboratory values, cardiac functional indices (e.g., left ventricular ejection fraction, atrial dimensions), operative time, and length of chest drainage. Categorical variables included sex, education level, marital status, hypertension status, surgical approach, extent of resection, tumour characteristics, and medication history.

### 2.4 Data preprocessing

Prior to model development, data preprocessing was performed to handle missing values and prepare variables for analysis. Continuous variables were kept on their original scale, and any missing values were imputed using the median value derived from the training cohort. Categorical variables were encoded as dummy variables (one-hot encoding). If a categorical predictor had missing entries, a separate “Unknown” category was created to retain those cases without introducing bias, and to avoid excessive data loss. All preprocessing steps (imputation and encoding) were performed using only the training set, and then applied to the test set, to prevent data leakage. The same final set of predictors was used for all models to ensure comparability of performance.

### 2.5 Dataset splitting

The cohort of 540 patients was randomly divided into a training set (*n* = 379) and an independent test set (*n* = 161) using a fixed random seed to ensure reproducibility. The training cohort was used exclusively for feature selection and model development, whereas the test cohort was reserved for independent performance evaluation and validation.

### 2.6 Feature selection using LASSO regression

**Figure 2:**
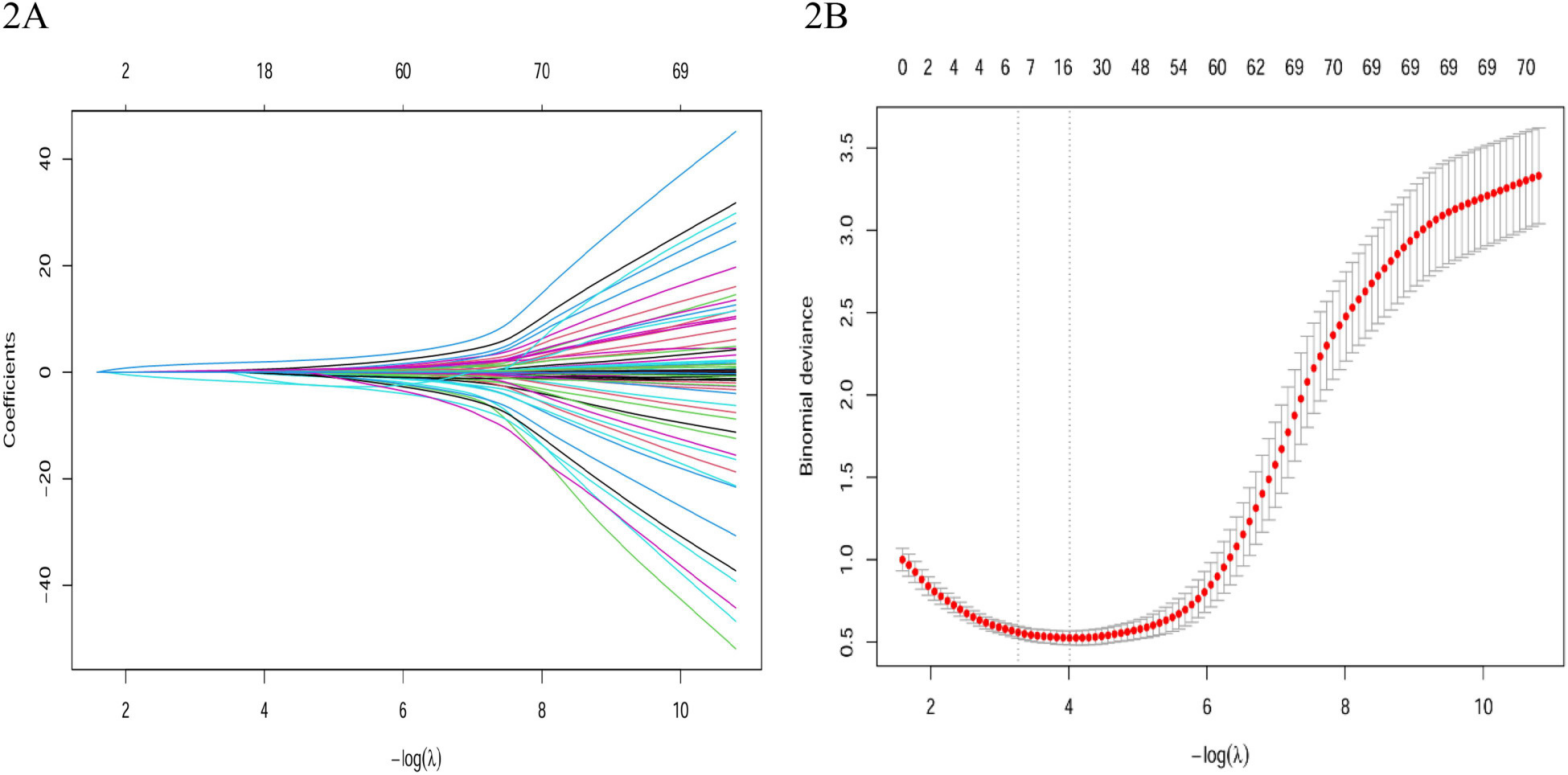
Feature selection using least absolute shrinkage and selection operator (LASSO) regression in the training cohort. **(A)** LASSO coefficient profiles for candidate predictors plotted against −log(*λ*). As the penalty increased, most coefficients were shrunk toward zero. **(B)** Ten-fold cross-validation curve for selection of the optimal *λ* based on binomial deviance. The two vertical dashed lines indicate *λ*_min (minimum mean cross-validated deviance) and *λ*_1se (the largest *λ* within one standard error of the minimum). *λ*_1se was selected to derive a parsimonious and stable predictor set.

To identify the most informative predictors while minimising overfitting, feature selection was performed with LASSO regression on the training cohort. After dummy encoding of categorical variables, a 10-fold cross-validation procedure (with a fixed fold assignment and random seed for reproducibility) was run to determine the optimal regularisation parameter (*λ*) based on the binomial deviance. We selected the largest *λ* within one standard error of the minimum deviance (the *λ*_1se criterion) to derive a parsimonious and stable feature set. Predictors with non-zero coefficients at *λ*_1se were retained for subsequent model development. This procedure yielded six predictors: age, education level, hypertension, marital status, postoperative pain score, and surgical approach (Figures 2A,B shows the LASSO coefficient profiles and cross-validation curve for *λ* selection.)

### 2.7 Development of machine-learning models

Using the six predictors selected by LASSO, we developed seven ML models in the training cohort: LR, KNN, DT, RF, XGBoost, SVM, and NN. All models were trained on the same training data and predictor set for fair comparison. Hyperparameters for each model were tuned within the training cohort via internal cross-validation (grid search or heuristic tuning as appropriate). We did not employ a fully nested cross-validation approach for hyperparameter optimisation, as our primary objective was to compare models and translate the best-performing model into a clinical tool rather than to exhaustively fine-tune each algorithm.

### 2.8 Model performance evaluation

Model performance was evaluated in both the training and test cohorts using multiple metrics, including AUC, overall accuracy, precision (positive predictive value), recall (sensitivity), and F1 score. Predicted probability outputs from each model were used to generate receiver operating characteristic (ROC) curves, assess calibration, and perform decision curve analysis, as described below.

### 2.9 Calibration assessment

Calibration performance was assessed using calibration plots, which compare the predicted probabilities with observed outcome frequencies across predefined risk strata. A 45-degree line on the plot represents perfect calibration (predicted risk equal to observed risk). Calibration curves were generated separately for the training and test cohorts for each model to evaluate how closely the model's risk predictions matched actual outcomes.

### 2.10 Clinical utility assessment

The clinical usefulness of each prediction model was evaluated with decision curve analysis (DCA) in the independent test cohort. DCA quantifies the net benefit of using a model to guide decisions (e.g., prophylactic treatment) across a range of threshold probabilities, compared against default strategies of treating all patients vs. treating none. For each model, we plotted the net benefit across relevant threshold probability values. A model is considered clinically useful if its net benefit curve lies above those of the “treat-all” and “treat-none” strategies within a reasonable range of threshold probabilities.

### 2.11 Nomogram construction

Considering the overall model performances (discrimination, calibration, and net benefit), we selected the LR model as the final model for clinical translation. Although some complex ML models achieved excellent apparent performance in training, their test performance deteriorated more markedly, indicating potential overfitting. In contrast, LR provided robust performance and easier interpretation. We therefore constructed a nomogram based on the LR model to provide individualised risk estimation of POAF. Each of the six predictors retained by LASSO was assigned a scaled number of points according to its regression coefficient in the LR model. By summing the points for a given patient's profile, a total score is obtained, which can be mapped to that patient's predicted probability of POAF on the nomogram (Figure 6 illustrates the nomogram for POAF risk prediction.)

### 2.12 Statistical analysis

Baseline characteristics were summarised using appropriate descriptive statistics. Continuous variables were reported as mean ± standard deviation (SD) and compared between groups using Student's *t*-test or the Mann–Whitney *U*-test, as appropriate based on normality of distribution. Categorical variables were expressed as counts and percentages and compared using the *χ*^2^ test or Fisher's exact test when expected cell counts were low. For model development, the training set was used for all feature selection and model training, and the test set was used only for evaluating final model performance. The primary discrimination metric for each model was the AUC, with higher AUC indicating better differentiation between patients with and without POAF. We also calculated accuracy, precision, recall, and F1 score to provide a comprehensive assessment of classification performance in both cohorts. ROC curves were generated for each model in both the training and test sets to visualise discriminative ability. Model calibration was assessed by plotting predicted vs. observed event rates and examining how closely the curves followed the 45° reference line. Clinical utility was evaluated via DCA, as described above, by comparing net benefits. All statistical analyses and model training were performed using R software (version 4.4.2). Key R packages used included glmnet, caret, pROC, rms, rmda, ggplot2, randomForest, xgboost, e1071, and nnet. All significance tests were two-sided, and a *P* value < 0.05 was considered statistically significant.

## 3 Results

### 3.1 Study population and baseline characteristics

A total of 540 patients undergoing lung cancer surgery met the inclusion criteria, of whom 107 (19.8%) developed POAF during the postoperative hospital stay. The cohort was randomly divided into a training set (*n* = 379) and a test set (*n* = 161). Table 1 presents the baseline characteristics stratified by POAF occurrence and by cohort. Patients who developed POAF were older on average than those without POAF (*P* < 0.001) and had a higher prevalence of hypertension (*P* < 0.001) and prior cardiac disease (*P* < 0.001). In terms of perioperative factors, an open thoracotomy approach was much more common in the POAF group, whereas video-assisted thoracoscopic surgery (VATS) predominated in the non-POAF group (*P* < 0.001). Patients with POAF also reported higher postoperative pain scores (*P* < 0.001), indicating more severe pain or analgesia needs. There were also differences in certain cardiac functional indices: patients with POAF had slightly lower left ventricular ejection fraction (LVEF) and larger left atrial dimensions/volume (all *P* < 0.05) compared to those without POAF. Some inflammatory markers differed between groups as well (e.g., higher neutrophil-to-lymphocyte ratio and lower absolute lymphocyte count in POAF patients, *P* < 0.01), suggesting a greater inflammatory response in those who developed arrhythmia. Other demographic, tumour-related, pulmonary function, and laboratory variables were generally similar between the POAF and non-POAF groups. When comparing the training and test cohorts, their baseline characteristics were largely well-matched, with no significant differences in most variables, except that the distribution of education levels, certain medication categories, and postoperative drainage duration showed some variation between the two cohorts.

**Table 1:** Baseline characteristics of patients with and without postoperative atrial fibrillation and comparison between the training and test cohorts.

Variable	Level	Non-POAF (*n* = 433)	POAF(*n* = 107)	*p*	Training (*n* = 379)	Test (*n* = 161)	*p*
Sex (%)	Female	147 (33.9)	39 (36.4)	0.709	133 (35.1)	53 (32.9)	0.699
	Male	286 (66.1)	68 (63.6)		246 (64.9)	108 (67.1)	
Marital status (%)	Married	347 (80.1)	66 (61.7)	<0.001	291 (76.8)	122 (75.8)	0.888
	Others	86 (19.9)	41 (38.3)		88 (23.2)	39 (24.2)	
Residence (%)	Urban	183 (42.3)	47 (43.9)	0.951	159 (42.0)	71 (44.1)	0.898
	Town	122 (28.2)	29 (27.1)		107 (28.2)	44 (27.3)	
	Rural	128 (29.6)	31 (29.0)		113 (29.8)	46 (28.6)	
Education (%)	Primary or below	106 (24.5)	30 (28.0)	0.042	83 (21.9)	53 (32.9)	0.041
	Junior or Vocational	146 (33.7)	24 (22.4)		129 (34.0)	41 (25.5)	
	Senior high	96 (22.2)	35 (32.7)		93 (24.5)	38 (23.6)	
	College or above	85 (19.6)	18 (16.8)		74 (19.5)	29 (18.0)	
Occupation (%)	Manual	133 (30.7)	34 (31.8)	0.758	112 (29.6)	55 (34.2)	0.529
	Non manual	91 (21.0)	19 (17.8)		81 (21.4)	29 (18.0)	
	Retired	160 (37.0)	44 (41.1)		147 (38.8)	57 (35.4)	
	Others	49 (11.3)	10 (9.3)		39 (10.3)	20 (12.4)	
Tumor type (%)	NSCLC	378 (87.3)	94 (87.9)	0.822	326 (86.0)	146 (90.7)	0.258
	SCLC	33 (7.6)	9 (8.4)		34 (9.0)	8 (5.0)	
	Others	22 (5.1)	4 (3.7)		19 (5.0)	7 (4.3)	
Tumor stage (%)	I	206 (47.6)	58 (54.2)	0.59	189 (49.9)	75 (46.6)	0.235
	II	118 (27.3)	28 (26.2)		96 (25.3)	50 (31.1)	
	III	87 (20.1)	17 (15.9)		72 (19.0)	32 (19.9)	
	IV	22 (5.1)	4 (3.7)		22 (5.8)	4 (2.5)	
Treatment (%)	Surgery only	240 (55.4)	62 (57.9)	0.751	206 (54.4)	96 (59.6)	0.371
	Surgery plus adjuvant	145 (33.5)	32 (29.9)		132 (34.8)	45 (28.0)	
	Neoadjuvant plus surgery	40 (9.2)	12 (11.2)		36 (9.5)	16 (9.9)	
	Palliative	8 (1.8)	1 (0.9)		5 (1.3)	4 (2.5)	
Medication (%)	None	155 (35.8)	44 (41.1)	0.269	125 (33.0)	74 (46.0)	0.006
	Antibiotic	101 (23.3)	21 (19.6)		83 (21.9)	39 (24.2)	
	Beta blocker	60 (13.9)	21 (19.6)		68 (17.9)	13 (8.1)	
	Antiarrhythmic	59 (13.6)	12 (11.2)		51 (13.5)	20 (12.4)	
	Multiple	58 (13.4)	9 (8.4)		52 (13.7)	15 (9.3)	
Surgical approach (%)	VATS	340 (78.5)	30 (28.0)	<0.001	266 (70.2)	104 (64.6)	0.239
	Open thoracotomy	93 (21.5)	77 (72.0)		113 (29.8)	57 (35.4)	
Surgical site (%)	Left	222 (51.3)	45 (42.1)	0.11	187 (49.3)	80 (49.7)	1
	Right	211 (48.7)	62 (57.9)		192 (50.7)	81 (50.3)	
Resection extent (%)	Wedge	66 (15.2)	20 (18.7)	0.119	56 (14.8)	30 (18.6)	0.507
	Segmentectomy	104 (24.0)	16 (15.0)		84 (22.2)	36 (22.4)	
	Lobectomy	263 (60.7)	71 (66.4)		239 (63.1)	95 (59.0)	
Anesthesia (%)	GA	364 (84.1)	96 (89.7)	0.186	326 (86.0)	134 (83.2)	0.483
	GA plus regional	69 (15.9)	11 (10.3)		53 (14.0)	27 (16.8)	
Analgesia (%)	IV_PCA	202 (46.7)	44 (41.1)	0.328	170 (44.9)	76 (47.2)	0.835
	Epidural PCA	70 (16.2)	24 (22.4)		64 (16.9)	30 (18.6)	
	Oral	98 (22.6)	27 (25.2)		91 (24.0)	34 (21.1)	
	Regional block	63 (14.5)	12 (11.2)		54 (14.2)	21 (13.0)	
PFT (%)	Normal	241 (55.7)	68 (63.6)	0.32	221 (58.3)	88 (54.7)	0.688
	Mild abnormal	138 (31.9)	27 (25.2)		114 (30.1)	51 (31.7)	
	Moderate abnormal	54 (12.5)	12 (11.2)		44 (11.6)	22 (13.7)	
Smoking (%)	No	260 (60.0)	79 (73.8)	0.011	239 (63.1)	100 (62.1)	0.911
	Yes	173 (40.0)	28 (26.2)		140 (36.9)	61 (37.9)	
Alcohol (%)	No	307 (70.9)	65 (60.7)	0.056	258 (68.1)	114 (70.8)	0.599
	Yes	126 (29.1)	42 (39.3)		121 (31.9)	47 (29.2)	
Hypertension (%)	No	354 (81.8)	29 (27.1)	<0.001	272 (71.8)	111 (68.9)	0.577
	Yes	79 (18.2)	78 (72.9)		107 (28.2)	50 (31.1)	
Diabetes (%)	No	351 (81.1)	87 (81.3)	1	304 (80.2)	134 (83.2)	0.484
	Yes	82 (18.9)	20 (18.7)		75 (19.8)	27 (16.8)	
CAD (%)	No	373 (86.1)	85 (79.4)	0.114	323 (85.2)	135 (83.9)	0.783
	Yes	60 (13.9)	22 (20.6)		56 (14.8)	26 (16.1)	
Thyroid disease (%)	No	395 (91.2)	98 (91.6)	1	344 (90.8)	149 (92.5)	0.614
	Yes	38 (8.8)	9 (8.4)		35 (9.2)	12 (7.5)	
Cardiac history (%)	No	396 (91.5)	80 (74.8)	<0.001	334 (88.1)	142 (88.2)	1
	Yes	37 (8.5)	27 (25.2)		45 (11.9)	19 (11.8)	
Pulmonary history (%)	No	367 (84.8)	92 (86.0)	0.868	324 (85.5)	135 (83.9)	0.722
	Yes	66 (15.2)	15 (14.0)		55 (14.5)	26 (16.1)	
Malignancy history (%)	No	401 (92.6)	98 (91.6)	0.878	352 (92.9)	147 (91.3)	0.65
	Yes	32 (7.4)	9 (8.4)		27 (7.1)	14 (8.7)	
Metastasis (%)	No	401 (92.6)	101 (94.4)	0.664	347 (91.6)	155 (96.3)	0.076
	Yes	32 (7.4)	6 (5.6)		32 (8.4)	6 (3.7)	
Robotic (%)	No	377 (87.1)	95 (88.8)	0.751	326 (86.0)	146 (90.7)	0.176
	Yes	56 (12.9)	12 (11.2)		53 (14.0)	15 (9.3)	
LN dissection (%)	No	134 (30.9)	36 (33.6)	0.673	127 (33.5)	43 (26.7)	0.146
	Yes	299 (69.1)	71 (66.4)		252 (66.5)	118 (73.3)	
PCA (%)	No	161 (37.2)	39 (36.4)	0.977	145 (38.3)	55 (34.2)	0.421
	Yes	272 (62.8)	68 (63.6)		234 (61.7)	106 (65.8)	
FEV1 [mean (SD)]		2.05 (0.57)	2.09 (0.52)	0.505	2.06 (0.57)	2.06 (0.53)	0.97
FEV1_FVC [mean (SD)]		71.51 (8.79)	71.76 (7.61)	0.785	71.32 (8.78)	72.13 (8.02)	0.315
BMI [mean (SD)]		23.71 (3.16)	23.21 (3.04)	0.142	23.69 (3.15)	23.44 (3.11)	0.404
Baseline HR [mean (SD)]		78.99 (10.83)	81.31 (10.06)	0.045	79.50 (10.33)	79.32 (11.58)	0.858
LVEF [mean (SD)]		60.45 (6.03)	58.83 (5.03)	0.011	60.31 (5.83)	59.68 (5.98)	0.249
LA_AP [mean (SD)]		36.99 (4.32)	38.26 (4.70)	0.008	37.39 (4.40)	36.88 (4.47)	0.221
LA_volume [mean (SD)]		36.51 (9.53)	40.30 (9.97)	<0.001	37.06 (9.56)	37.75 (10.15)	0.447
NLR [mean (SD)]		2.99 (1.64)	3.53 (1.99)	0.004	3.11 (1.68)	3.05 (1.83)	0.717
PLR [mean (SD)]		1.19 (0.66)	1.31 (0.66)	0.073	1.21 (0.68)	1.20 (0.63)	0.853
WBC [mean (SD)]		6.47 (1.44)	6.34 (1.65)	0.424	6.40 (1.47)	6.53 (1.52)	0.369
NEUT [mean (SD)]		4.08 (1.37)	4.30 (1.39)	0.152	4.15 (1.34)	4.06 (1.44)	0.475
LYMPH [mean (SD)]		1.53 (0.45)	1.39 (0.43)	0.005	1.50 (0.45)	1.50 (0.45)	0.905
IL6 [mean (SD)]		7.79 (4.63)	8.54 (4.66)	0.136	8.27 (4.83)	7.17 (4.08)	0.012
IL8 [mean (SD)]		10.72 (5.46)	11.00 (5.61)	0.636	10.59 (5.54)	11.21 (5.36)	0.233
TNFa [mean (SD)]		5.01 (2.38)	5.19 (2.29)	0.474	5.06 (2.41)	5.01 (2.26)	0.844
IL1 [mean (SD)]		2.51 (1.38)	2.44 (1.42)	0.623	2.52 (1.38)	2.42 (1.41)	0.439
CRP [mean (SD)]		9.97 (8.49)	10.33 (8.93)	0.691	10.12 (8.03)	9.85 (9.76)	0.734
BNP [mean (SD)]		75.66 (58.46)	87.82 (54.03)	0.051	75.54 (54.94)	84.04 (63.73)	0.118
K [mean (SD)]		4.12 (0.35)	4.11 (0.32)	0.817	4.12 (0.34)	4.10 (0.36)	0.484
Na [mean (SD)]		139.00 (2.93)	139.20 (3.23)	0.532	138.99 (3.03)	139.15 (2.88)	0.559
Ca [mean (SD)]		2.24 (0.10)	2.22 (0.10)	0.086	2.24 (0.10)	2.24 (0.10)	0.907
Mg [mean (SD)]		0.86 (0.10)	0.87 (0.09)	0.538	0.86 (0.10)	0.86 (0.11)	0.691
Albumin [mean (SD)]		37.76 (4.48)	37.55 (4.77)	0.674	37.70 (4.56)	37.76 (4.50)	0.906
Prealbumin [mean (SD)]		209.24 (59.59)	213.62 (65.68)	0.505	209.61 (59.26)	211.26 (64.47)	0.773
NRS [mean (SD)]		2.02 (1.42)	2.20 (1.53)	0.26	2.04 (1.42)	2.09 (1.49)	0.693
Age [mean (SD)]		61.16 (8.76)	68.09 (7.13)	<0.001	62.81 (8.99)	61.89 (8.68)	0.276
Disease_duration [mean (SD)]		7.65 (5.43)	8.43 (6.34)	0.197	7.50 (5.35)	8.51 (6.21)	0.057
Pain score [mean (SD)]		2.22 (1.27)	3.43 (1.57)	<0.001	2.49 (1.43)	2.38 (1.39)	0.392
Operation time [mean (SD)]		180.55 (44.32)	188.02 (44.91)	0.12	182.64 (44.80)	180.59 (43.89)	0.625
Drainage time [mean (SD)]		4.35 (1.74)	4.52 (1.72)	0.359	4.28 (1.74)	4.63 (1.71)	0.031
Surgeon experience [mean (SD)]		9.00 (3.93)	9.45 (3.85)	0.294	9.04 (3.92)	9.21 (3.90)	0.647
LOS [mean (SD)]		8.19 (2.75)	7.76 (3.05)	0.157	8.02 (2.76)	8.29 (2.92)	0.306

POAF, postoperative atrial fibrillation; VATS, video-assisted thoracoscopic surgery; GA, general anesthesia; PCA, patient-controlled analgesia; PFT, pulmonary function test; FEV1, forced expiratory volume in 1s; FVC, forced vital capacity; LVEF, left ventricular ejection fraction; LA_AP, left atrial anteroposterior diameter; NLR, neutrophil-to-lymphocyte ratio; PLR, platelet-to-lymphocyte ratio; WBC, white blood cell count; NEUT, neutrophil count; LYMPH, lymphocyte count; IL, interleukin; TNF*α*, tumor necrosis factor-α; CRP, C-reactive protein; BNP, B-type natriuretic peptide; NRS, numeric rating scale; LOS, length of stay; CAD, coronary artery disease.

Data are presented as *n* (%) for categorical variables and mean (SD) for continuous variables. *P* values were calculated using the chi-square test or Fisher's exact test for categorical variables, and Student's t test (or Wilcoxon rank-sum test, as appropriate) for continuous variables.

### 3.2 Feature selection with LASSO

LASSO regression was applied in the training cohort to select the most informative predictors while reducing overfitting. After full dummy-variable encoding of all categorical features, we performed 10-fold cross-validation to determine the optimal degree of penalisation. Figure 2A shows the LASSO coefficient trajectories for all candidate predictors as the regularisation penalty (*λ*) increases—most coefficients shrink towards zero with stronger penalisation. Figure 2B depicts the cross-validation curve used to select *λ*: we chose the *λ*_1se value (the largest *λ* within one standard error of the minimum deviance) to favour a simpler model. Using this criterion, six predictors had non-zero coefficients at *λ*_1se and were thus retained: age, education level, hypertension, marital status, postoperative pain score, and surgical approach. These six features were carried forward for subsequent model development and for constructing the nomogram. BNP was included as a clinically relevant candidate predictor in the initial feature-selection process. However, its coefficient was shrunk to zero under the *λ*_1se criterion and therefore BNP was not retained in the final parsimonious model.

### 3.3 Model development and performance comparison

Using the six LASSO-selected predictors, we trained seven different models in the training cohort: LR, KNN, DT, RF, XGBoost, SVM, and NN. Performance of all models was then evaluated in the independent test cohort. Table 2 summarises the discrimination and classification metrics for each model in both the training and test sets (including AUC, accuracy, precision, recall, and F1 score). In the training cohort, most models showed strong discrimination (AUC 0.83–0.99). Notably, the RF and NN models achieved very high AUCs approaching 1.0 in training, along with high accuracy; however, such near-perfect performance on training data suggests that these complex models may be overfitting to the idiosyncrasies of the training set. Other models, including LR, also had excellent training AUCs but with a simpler model structure. In the independent test cohort, overall model performance declined to varying degrees for all algorithms, indicating the more realistic performance on unseen data. The LR model maintained the highest and most stable performance on the test set (AUC = 0.855; accuracy = 0.857; precision = 0.667; recall = 0.563; F1 = 0.610). KNN and XGBoost achieved similarly high test AUCs (0.849 and 0.852, respectively), but XGBoost in particular showed a much lower recall (0.344), indicating it missed a large proportion of true POAF cases (lower sensitivity). The simpler KNN model had moderate recall (0.563) and precision (0.692) on the test set, comparable to LR. By contrast, the more complex DT, RF, SVM, and NN models all yielded lower AUCs in the test cohort (ranging ∼0.77–0.83) and imbalanced precision-recall profiles (for example, the NN had precision ∼0.48 and recall ∼0.50 in test, indicating poor positive predictive value despite moderate sensitivity). These results suggest that the complex nonlinear models did not generalise as well to new data, likely due to overfitting, whereas the logistic regression—despite its simplicity—generalised better.

**Table 2:** Performance comparison of machine-learning models for prediction of postoperative atrial fibrillation.

Dataset	Model	AUC	Accuracy	Precision	Recall	F1
Train	LR	0.9477	0.8945	0.7869	0.64	0.7059
Test	LR	0.8549	0.8571	0.6667	0.5625	0.6102
Train	KNN	0.9573	0.8971	0.8462	0.5867	0.6929
Test	KNN	0.8494	0.8634	0.6923	0.5625	0.6207
Train	DT	0.8272	0.8865	0.7424	0.6533	0.695
Test	DT	0.7672	0.8385	0.6	0.5625	0.5806
Train	RF	0.9945	0.9683	1	0.84	0.913
Test	RF	0.8308	0.8509	0.6538	0.5312	0.5862
Train	XGB	0.9645	0.905	0.9333	0.56	0.7
Test	XGB	0.8523	0.8447	0.7333	0.3438	0.4681
Train	SVM	0.9521	0.9129	0.8621	0.6667	0.7519
Test	SVM	0.8205	0.8509	0.6538	0.5312	0.5862
Train	NN	0.9702	0.9683	0.9701	0.8667	0.9155
Test	NN	0.813	0.795	0.4848	0.5	0.4923

AUC, area under the receiver operating characteristic curve; LR, logistic regression; KNN, k-nearest neighbors; DT, decision tree; RF, random forest; XGBoost, extreme gradient boosting; SVM, support vector machine; NN, neural network.

Model performance was evaluated in the training and independent test cohorts using AUC, accuracy, precision, recall, and F1 score.

**Figure 3:**
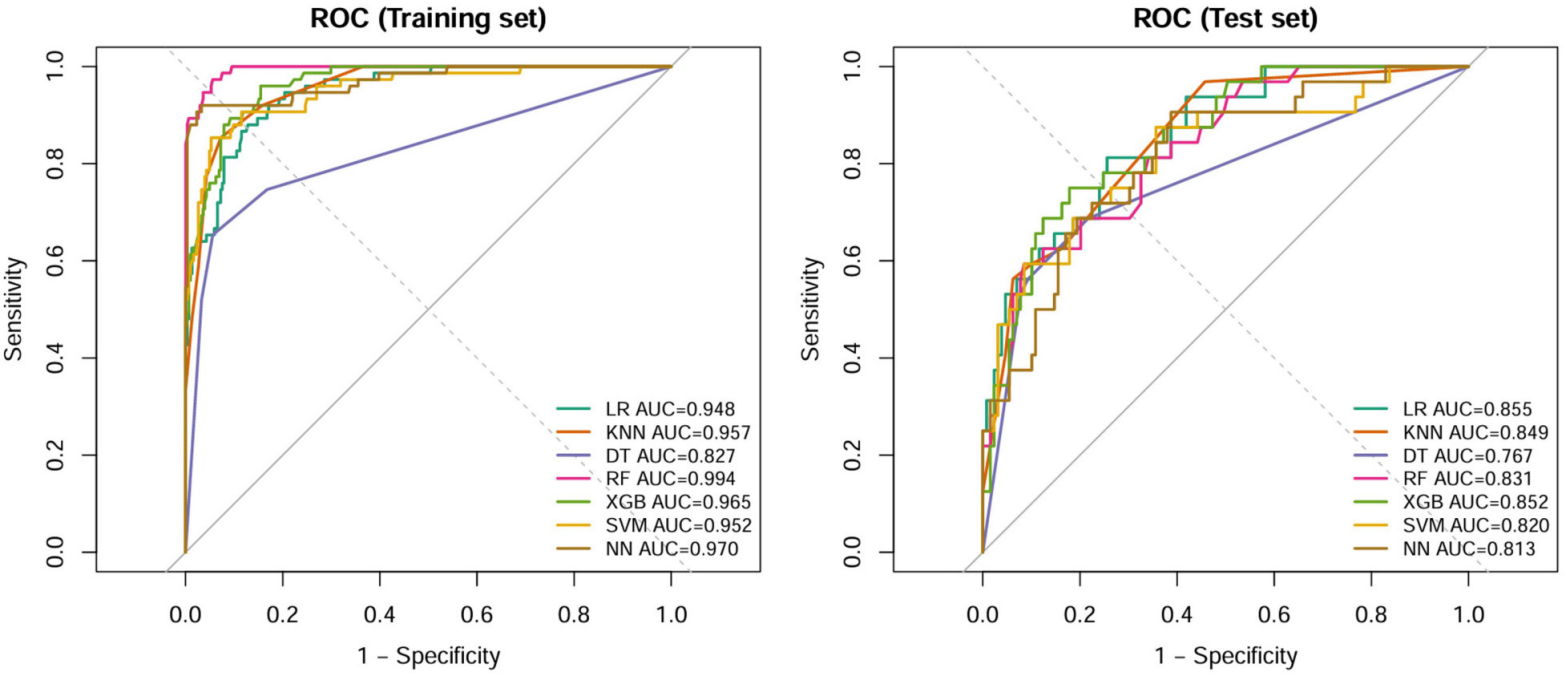
Receiver operating characteristic (ROC) curves of seven machine-learning models in the training and test cohorts. ROC curves illustrate the discriminative performance of logistic regression (LR), k-nearest neighbors (KNN), decision tree (DT), random forest (RF), extreme gradient boosting (XGBoost), support vector machine (SVM), and neural network (NN) in the training cohort (left) and the independent test cohort (right). The area under the ROC curve (AUC) for each model is shown in the legend.

Figure 3 shows the ROC curves for all seven models in both the training and test cohorts. In the training set (Figure 3, left panel), several nonlinear and ensemble models (RF, NN, XGBoost) achieved almost perfect discrimination (their ROC curves nearly reach the top-left corner), whereas the LR model also achieved a high AUC with a much simpler linear form. In the test cohort (Figure 3, right panel), the ROC curves for all models shifted closer to the diagonal line, reflecting reduced discrimination on new data. The LR model's ROC curve remained the highest overall, indicating the best trade-off of sensitivity and specificity. Models that had nearly perfect ROC in training (RF, NN) showed the greatest drops in the test ROC, consistent with limited generalisability of those models.

### 3.4 Calibration performance of the models

**Figure 4:**
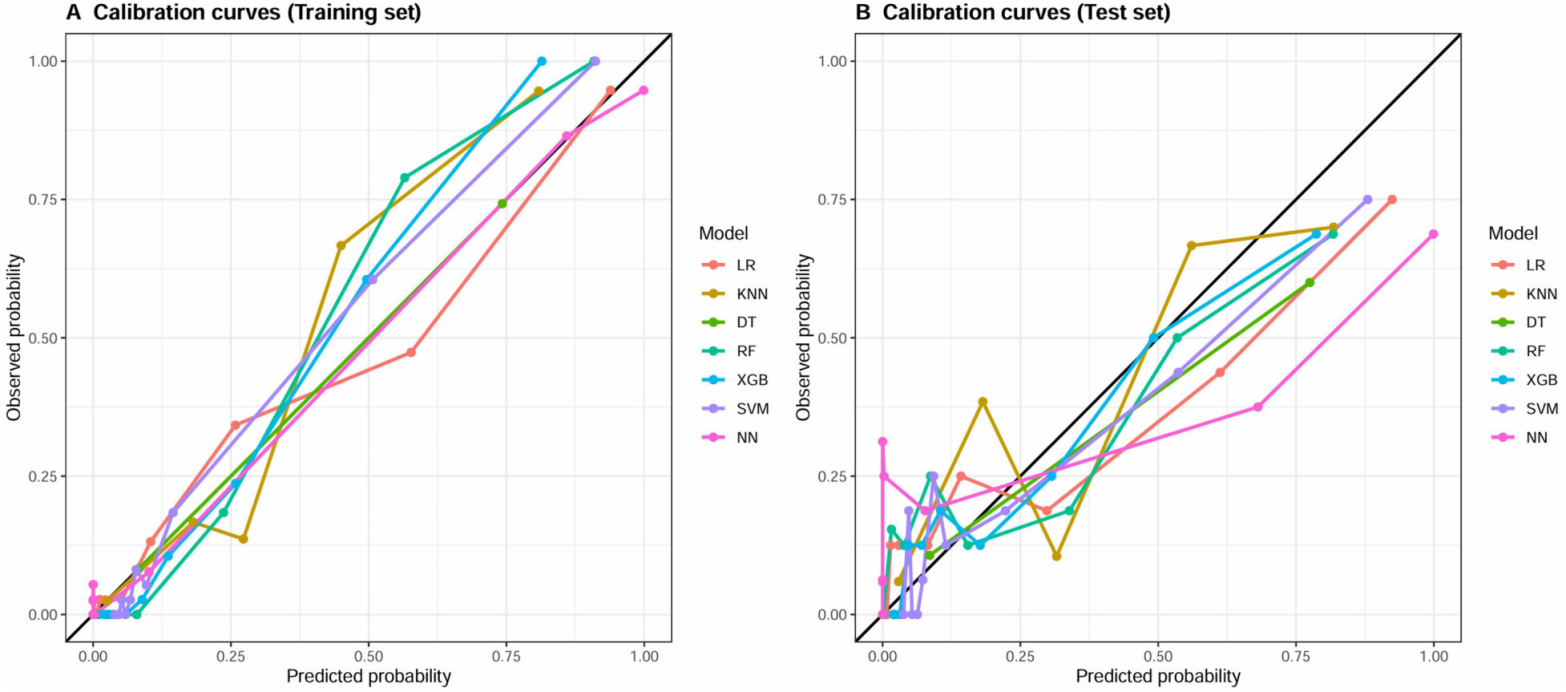
Calibration curves of the machine-learning models in the training and test cohorts. Calibration curves depict the agreement between predicted probabilities and observed POAF outcomes for each model in the training cohort **(A)** and test cohort **(B)**. The diagonal line represents perfect calibration (predicted risk equals observed risk). Curves closer to the diagonal indicate better calibration.

The calibration of the seven prediction models was assessed using calibration curves in both the training and test cohorts (Figure 4). Calibration refers to the agreement between predicted probabilities and observed outcomes across different risk levels. In the training cohort, most models appeared reasonably calibrated, with predicted probabilities closely aligned with the ideal 45° reference line. LR, KNN, XGBoost, and SVM showed good agreement between predicted and observed risks across the majority of probability ranges. In contrast, some deviation from perfect calibration was observed for the RF and NN models at higher predicted risk levels, indicating potential overestimation of risk despite their high apparent discrimination. In the independent test cohort, overall calibration performance declined modestly for all models. LR maintained relatively good calibration, with predictions remaining close to the diagonal reference line over low-to-moderate risk ranges. KNN and XGBoost exhibited acceptable calibration in test, though they showed greater variability at lower predicted probability levels. By contrast, NN and RF demonstrated more pronounced deviations from ideal calibration in the test cohort, particularly at the extremes of predicted risk, suggesting reduced reliability of their absolute risk estimates.

### 3.5 Clinical utility via decision curve analysis

**Figure 5:**
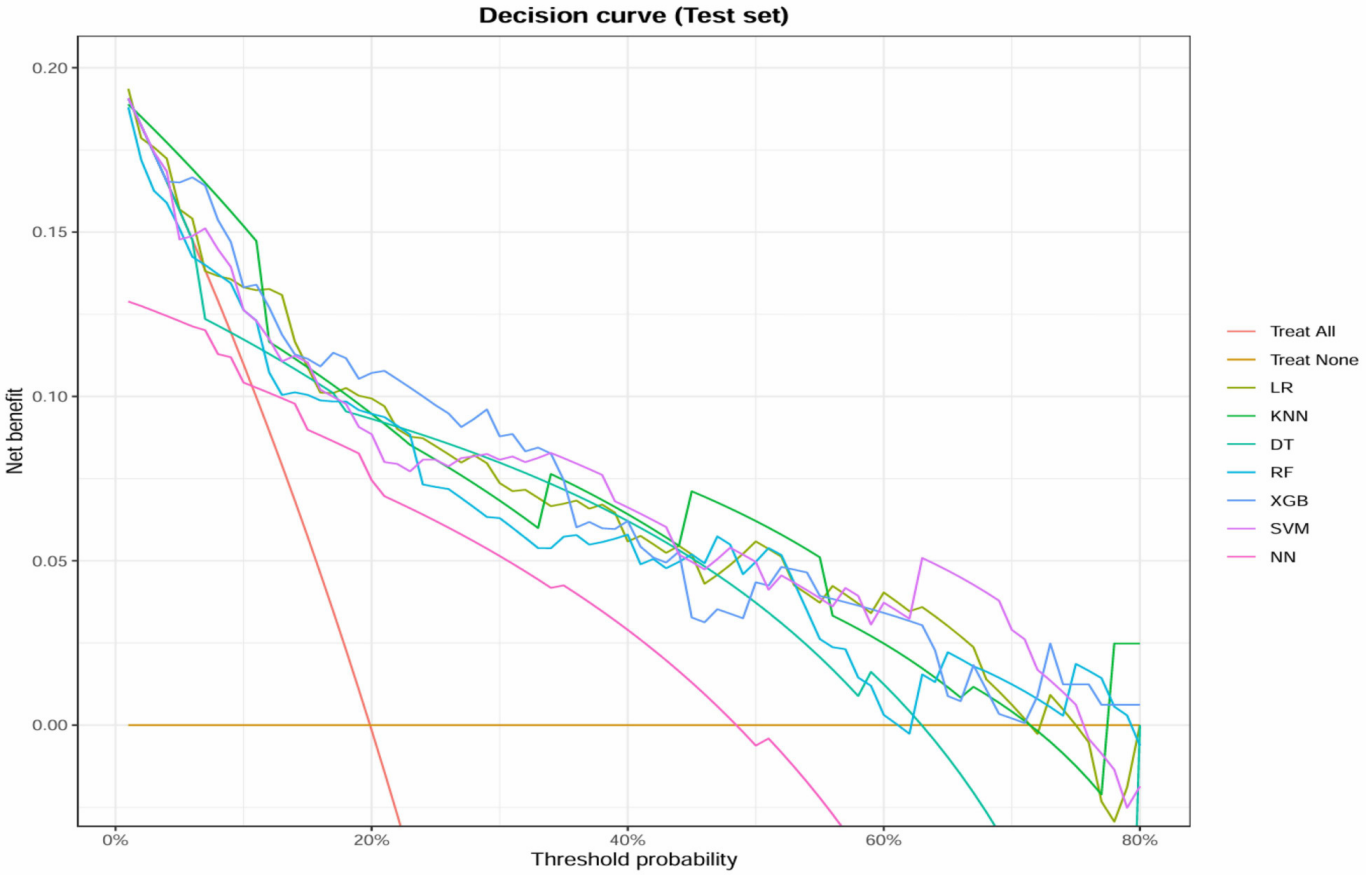
Decision curve analysis (DCA) of machine-learning models in the test cohort. Decision curves show the net benefit of each prediction model across a range of threshold probabilities in the independent test cohort. The horizontal line represents the “treat-none” strategy, and the oblique line represents the “treat-all” strategy. A model is considered clinically useful when it provides a higher net benefit than both default strategies over clinically relevant thresholds.

To evaluate the potential clinical value of each model, we performed DCA using the test cohort. Figure 5 illustrates the net benefit curves for the models across a range of threshold probabilities (the probability at which one might choose to intervene for POAF prevention). The horizontal line at net benefit = 0 corresponds to treating no patients (assuming none will get POAF), and the sloped line corresponds to treating all patients (assuming everyone is high-risk). A model is useful if its curve is above both lines in the range of thresholds of interest. As shown in Figure 5, across a wide range of reasonable threshold probabilities (for example, 5% to 40%), most of the models provided a higher net benefit than the treat-all or treat-none strategies, indicating that using any of the models to guide selective intervention would be better than intervening on all or none. Among the models, the LR model's net benefit curve was consistently among the highest. LR provided a clear net benefit advantage especially in the low-to-moderate threshold range (e.g., 10%–30% risk thresholds, which are clinically relevant for deciding prophylaxis). Some ML models (like XGBoost or KNN) had portions of their curves that were comparable to LR or even slightly higher at certain thresholds, but their net benefit tended to fluctuate. In contrast, LR maintained a stable, favourable net benefit across the full range, reflecting its robust and balanced performance. These DCA results suggest that the LR model would confer the greatest clinical benefit in guiding interventions to prevent POAF, as it yields fewer unnecessary interventions while still capturing high-risk patients, compared to the alternatives.

### 3.6 Development of the nomogram for POAF prediction

**Figure 6:**
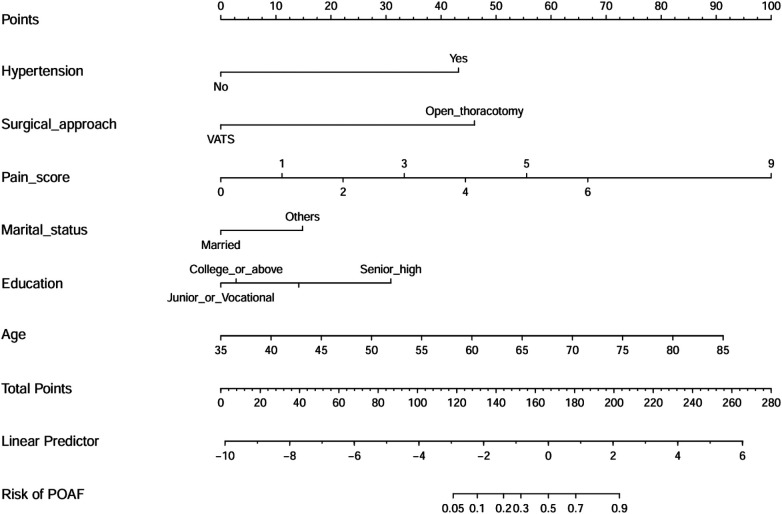
Nomogram for individualized prediction of postoperative atrial fibrillation. A nomogram integrating age, hypertension, surgical approach, postoperative pain score, marital status, and education level to estimate an individual's risk of POAF. For each predictor, points are assigned according to its value; the total points correspond to the patient's predicted probability of POAF.

Considering the overall performance across discrimination, calibration, and clinical utility, we selected LR as the final model for presentation and clinical translation. Although several machine-learning models achieved excellent apparent performance in the training cohort, their discrimination and calibration declined substantially on the independent test cohort, indicative of overfitting and limited generalizability. By contrast, LR demonstrated the most stable discrimination, acceptable calibration, and a consistently favorable net benefit across clinically relevant threshold probabilities. Based on the six variables selected by LASSO, an LR-based nomogram was constructed to facilitate individualized POAF risk prediction (Figure 6). The nomogram incorporates six predictors: age, hypertension status, surgical approach, postoperative pain score, marital status, and education level. Each predictor is assigned a weighted number of points according to its regression coefficient in the LR model. By summing the points corresponding to a patient's profile, a total score is obtained, which can be mapped to the estimated probability of POAF. As illustrated in Figure 6, older age, the presence of hypertension, an open thoracotomy (vs. VATS), higher postoperative pain score, unmarried status, and lower education level are each associated with an increased predicted risk of POAF. The nomogram provides a convenient and intuitive bedside tool for risk estimation, which may facilitate perioperative risk stratification and personalized management. In particular, the nomogram may be most useful during the early postoperative period, when clinical decisions regarding monitoring intensity and preventive strategies are actively made.

## 4 Discussion

In this study, we developed and validated a prediction model for POAF in patients undergoing lung cancer surgery using routinely available perioperative clinical data. By applying LASSO regression for feature selection and systematically comparing multiple ML algorithms, we identified logistic regression (LR) as the most robust and clinically applicable model in this context. We then constructed an interpretable nomogram based on the LR model to facilitate individualised POAF risk estimation at the point of care. This work contributes to thoracic surgical risk stratification by providing a POAF-focused tool tailored to lung cancer surgery patients. By focusing specifically on lung cancer surgery and prioritising both robustness and interpretability, this study complements existing POAF research and addresses a clinically relevant gap in thoracic surgical risk stratification. Importantly, this study emphasises that model robustness and clinical translatability may be more relevant than maximising apparent predictive accuracy in real-world perioperative settings when developing perioperative risk prediction tools.

The reported incidence of POAF after lung cancer surgery varies substantially across studies, reflecting marked heterogeneity in patient populations, surgical extent, and perioperative management. For example, Cardinale et al. (14) reported that the incidence of POAF after lung cancer surgery could be as high as 42%, particularly in patients undergoing extensive resections. In contrast, Ishibashi et al. (15) observed a lower incidence ranging from 6.4% to 12.6% following pulmonary lobectomy. Similarly, Ruan et al. (16) reported a POAF incidence of 8.38% in a cohort of patients with non–small cell lung cancer undergoing lung resection. These discrepancies across studies suggest that the incidence of POAF after lung cancer surgery is not a fixed value but varies substantially depending on surgical extent, patient characteristics, perioperative monitoring strategies, and study design. Notably, Tong et al. (17) reported an incidence of 2.6% for clinically important new-onset POAF in a large Chinese cohort. This incidence was substantially lower than that observed in the present study. However, this discrepancy should not be attributed to age alone, because the mean age of the two cohorts was broadly comparable. Several methodological and clinical differences may explain the higher incidence observed in our study. First, the outcome definition differed: Tong et al. focused on clinically important new-onset POAF, whereas our study recorded electrocardiographically documented POAF occurring during the postoperative hospital stay, including potentially transient or less symptomatic episodes. Second, the surgical case mix differed, with approximately one-third of our cohort undergoing open thoracotomy, which may be associated with greater surgical trauma, inflammatory response, postoperative pain, and sympathetic activation. Third, differences in postoperative rhythm monitoring duration and modality may influence the detection of silent or transient POAF. Therefore, direct comparison of POAF incidence across studies should be made cautiously, taking into account differences in outcome definition, monitoring strategy, surgical approach, and case mix. This further underscores the importance of developing institution-specific and procedure-specific risk prediction models rather than relying on a single universal incidence estimate.

The surgical approach was an important determinant: open thoracotomy was associated with a higher risk of POAF compared with VATS. This aligns with the general concept that greater surgical trauma and stress responses can increase arrhythmogenic triggers after lung resection (18). Postoperative pain score emerged as a notable predictor of POAF risk. Poor pain control may reflect heightened sympathetic activation and systemic stress (19), which can precipitate atrial arrhythmias; clinically, this finding reinforces the importance of optimising postoperative analgesia not only for comfort but potentially also for reducing cardiovascular complications. This finding is biologically plausible, as poorly controlled postoperative pain may amplify sympathetic activation, inflammatory responses, and autonomic imbalance, all of which have been implicated in the pathogenesis of atrial fibrillation. The retention of postoperative pain score in the final model highlights the value of incorporating early postoperative variables, which may capture dynamic physiological stress more effectively than static preoperative factors alone. This suggests that POAF risk is not solely determined preoperatively but continues to evolve in the early postoperative period, reinforcing the need for dynamic risk assessment. Therefore, the proposed nomogram should be understood as an early postoperative risk assessment tool, rather than a tool for purely preoperative prediction.

Inflammatory activation is biologically plausible in the pathogenesis of POAF, as surgical trauma, tissue injury, oxidative stress, and autonomic imbalance may promote atrial electrical instability. In the present cohort, some inflammatory markers, such as NLR, differed between patients with and without POAF, suggesting that systemic inflammation may be involved in POAF development. However, these markers were not retained in the final LASSO-selected model. This may indicate that their independent incremental predictive value was limited after accounting for other variables, such as surgical approach and postoperative pain score, which may indirectly capture the intensity of surgical trauma and postoperative inflammatory–sympathetic stress. In addition, inflammatory markers measured at a single perioperative time point may be influenced by multiple non-arrhythmic factors and may not fully reflect the dynamic inflammatory process preceding POAF. Therefore, the absence of inflammatory markers from the final model should not be interpreted as evidence against the role of inflammation in POAF pathophysiology. Future studies with serial inflammatory measurements may better clarify their temporal and incremental predictive value.

In addition, two sociodemographic variables—marital status and education level—were retained in the final model through LASSO feature selection. These variables should not be interpreted as direct mechanistic or pathophysiological determinants of POAF, as they do not provide a direct biological explanation for atrial electrical instability. Their retention may instead reflect contextual or care-related associations related to nuanced factors, such as differences in health literacy, symptom reporting, care-seeking behaviour, perioperative adherence, access to postoperative support, or other residual confounding (20). It is also possible that their predictive contribution partly reflects centre-specific patterns or statistical noise in this retrospective dataset. Therefore, these variables should be interpreted cautiously and should not be used in isolation for clinical decision-making. Their role in the model requires further assessment in external cohorts with different social and healthcare contexts.

Although several complex machine-learning models, including random forest, neural networks, and XGBoost, demonstrated excellent apparent performance in the training cohort, their discrimination and calibration deteriorated substantially in the independent test cohort. The near-perfect AUCs observed for some flexible algorithms in the training cohort should therefore be interpreted as apparent performance rather than evidence of true predictive superiority. In a moderate-sized dataset with a limited number of POAF events, highly flexible models may fit random noise, centre-specific practice patterns, or idiosyncratic correlations in the training data. The decline in performance in the independent test cohort suggests potential overfitting and reduced transportability even within the same institution. This finding reinforces the importance of evaluating not only discrimination but also calibration, clinical utility, and stability across datasets when selecting a model for clinical translation. This performance degradation persisted despite the use of cross-validation and standard hyperparameter tuning strategies, suggesting that model instability was driven by data characteristics rather than insufficient optimisation. Overall, these findings highlight an important methodological consideration in perioperative risk prediction. Postoperative atrial fibrillation after lung cancer surgery represents a multifactorial clinical outcome characterised by moderate event rates (21), heterogeneous perioperative influences, and a relatively high signal-to-noise ratio. This may explain why highly flexible algorithms showed reduced generalisability when applied to unseen patients. In contrast, the logistic regression model demonstrated consistently stable performance across training and test cohorts, with preserved discrimination, acceptable calibration, and favourable net benefit across clinically relevant threshold probabilities. In the decision curve analysis, the LR model provided greater net benefit than the treat-all and treat-none strategies across clinically relevant threshold probabilities, further supporting its potential clinical usefulness. This robustness is particularly relevant for clinical risk stratification, where reproducibility and reliability often outweigh marginal gains in apparent predictive accuracy. In surgical risk prediction, a modest reduction in apparent discrimination may be acceptable if accompanied by improved reproducibility, calibration, and clinical interpretability. From a methodological perspective, penalised regression-based approaches are well suited to moderate-sized clinical datasets with limited event counts, as they balance model complexity and variance while maintaining interpretability (22).

Importantly, the choice of logistic regression in this study was not merely a default or conservative option, but rather a deliberate decision aligned with the intended clinical application of the model. Risk prediction tools in thoracic surgery must support transparent decision-making, facilitate external validation, and enable bedside implementation. Compared with more complex algorithms, logistic regression offers clear advantages in interpretability, stability, and translational potential (23), particularly when the ultimate goal is to inform perioperative management rather than to maximise algorithmic performance in isolation. In particular, this nomogram may be most useful during the early postoperative period, when decisions regarding monitoring intensity and preventive strategies are actively made.

Recent studies have increasingly explored artificial intelligence and machine learning applications in thoracic surgery, including pulmonary nodule detection and characterisation, radiomics-based tumour classification, three-dimensional reconstruction, surgical planning, and postoperative outcome prediction (24). In parallel, artificial intelligence has also been increasingly applied in thoracic imaging, with emerging roles in image interpretation, lesion detection, quantitative imaging analysis, and clinical decision support (25). More specifically, AI-assisted endobronchial ultrasound has been evaluated for differentiating benign and malignant thoracic lymph nodes using pathological reference standards, providing a relevant example of how robust reference outcomes can support the interpretation of model performance and diagnostic generalizability (26). In addition, Ozcelik et al. (27) compared eight pre-trained convolutional neural network models for malignancy prediction using EBUS images with pathology-confirmed outcomes and reported stable training–testing performance for selected models, providing further context for interpreting model robustness, overfitting, and generalizability in clinically oriented AI studies. These studies highlight the potential of AI to integrate multidimensional clinical and imaging data to support diagnosis, staging, perioperative decision-making, and postoperative management. However, recent evidence also emphasises persistent methodological challenges, including heterogeneous model development, limited external validation, overfitting, insufficient interpretability, and variable clinical transferability. In this context, our findings are consistent with the broader literature suggesting that AI-based models in thoracic medicine should not be evaluated solely by apparent discrimination, but also by calibration, robustness, interpretability, and clinical utility. From a clinical translation perspective, the present findings should be interpreted with caution until the model is externally validated in independent cohorts. Although the LR-based nomogram showed more stable performance than more flexible algorithms, its generalizability to other institutions, surgical populations, and monitoring protocols remains uncertain. Future work should focus on external validation, prospective impact assessment, and integration into perioperative workflows to determine whether model-guided monitoring or preventive strategies can improve patient outcomes.

Several limitations should be acknowledged. First, this was a single-centre retrospective study with internal validation using a held-out test set, and no external validation cohort was available. Therefore, the present work should be interpreted as a derivation and internal validation study, rather than a fully externally validated prediction model. This represents a major limitation of the present study, as model performance may vary across institutions because of differences in patient characteristics, surgical practice, perioperative management, rhythm monitoring protocols, and data recording systems. Therefore, external validation in independent multicentre cohorts is required before the nomogram can be recommended for broader clinical implementation. In addition, the relatively high proportion of open thoracotomy in this cohort reflects the real-world surgical case mix at our institution during the study period. However, because VATS has become the predominant approach for early-stage lung cancer surgery in many contemporary settings, the generalizability of our model to populations in which minimally invasive surgery predominates may be limited. In particular, validation in cohorts with a higher proportion of VATS or robotic-assisted surgery is needed. Second, POAF was assessed during the postoperative hospital stay only. Although continuous electrocardiographic monitoring was routinely performed during the early postoperative period, monitoring intensity and duration may have varied according to patients' clinical condition and surgical risk. Therefore, asymptomatic POAF episodes occurring after discontinuation of continuous monitoring or after discharge may have been missed, potentially leading to underestimation of the true incidence. Third, some potentially important intraoperative variables were unavailable in this retrospective dataset. Detailed haemodynamic parameters, such as intraoperative hypotension, heart rate and blood pressure variability, estimated blood loss, fluid administration, transfusion, and vasoactive medication use, were not consistently recorded in a structured format and therefore could not be incorporated into the model. These variables may be strongly associated with perioperative physiological stress and POAF development. Their absence may limit the physiological grounding and mechanistic interpretability of the model. Finally, while we compared multiple algorithms, hyperparameter tuning for complex models was not exhaustive; performance could differ with larger datasets and more extensive optimisation. Future work should evaluate whether risk-guided preventive interventions, informed by this model, can improve outcomes. In addition, the clinical impact of implementing this model in routine practice was not evaluated and should be assessed in prospective impact studies.

Although BNP was included as a candidate predictor, it was not retained by LASSO feature selection under the *λ*_1se criterion. This may be partly related to its borderline between-group difference in the present cohort and the possibility that BNP did not provide sufficient incremental predictive information beyond the retained clinical and perioperative variables. In addition, NT-proBNP was not routinely available in this retrospective dataset and therefore could not be incorporated into the model. Future prospective studies with larger sample sizes and more complete biomarker assessment should further evaluate whether BNP or NT-proBNP can improve POAF risk prediction after lung cancer surgery.

Although we used the earliest available postoperative pain score recorded before POAF onset or diagnosis, the retrospective nature of the study may not fully eliminate residual temporal ambiguity between dynamic postoperative variables and POAF onset. Future prospective studies should standardise the timing of pain assessment, such as after recovery from anaesthesia or on postoperative day 1, before model implementation.

## 5 Conclusions

In conclusion, we have developed a robust and interpretable prediction model for POAF following lung cancer surgery. Compared to more complex ML algorithms, the logistic regression model demonstrated superior generalisability, ease of use, and clinical usefulness in this setting. We translated this model into a nomogram that provides surgeons and anesthesiologists with a practical tool for quantifying an individual patient's risk of POAF. This tool can aid in personalised perioperative decision-making, such as identifying patients who may benefit from enhanced monitoring or preventive therapies. Our study emphasizes that in this scenario, simpler may be better—a carefully validated and interpretable model can achieve superior generalisability and gain clinician trust. Before recommending routine clinical use of our nomogram, further external validation is required, and we encourage prospective studies to assess whether implementing such risk-guided strategies can indeed reduce POAF incidence or improve patient outcomes. With appropriate validation, this predictive tool has the potential to improve perioperative care by enabling early, individualized interventions for patients at risk of POAF.

## Data Availability

The datasets generated and/or analyzed during the current study are not publicly available due to privacy and ethical restrictions but are available from the corresponding author on reasonable request.
